# Perinatal tuberculosis: a diagnostic and treatment challenge in a remote area of Asmat Regency of South Papua, Indonesia (case report)

**DOI:** 10.11604/pamj.2022.42.303.36463

**Published:** 2022-08-23

**Authors:** Sitanaja Raymond, Helen Mayasari Subekti, Abed Ricky Hernando Sitompul

**Affiliations:** 1 Regional Public Hospital of Agats, Asmat Regency, South Papua, Indonesia,; 2 Department of Child Health, Regional Public Hospital of Agats, Asmat Regency, South Papua, Indonesia

**Keywords:** Perinatal tuberculosis, tuberculosis, neonate, perinatal, case report

## Abstract

Perinatal tuberculosis (TB) is a rare infectious disease. The diagnosis of perinatal TB is challenging due to its nonspecific clinical manifestations make it difficult to differentiate from other infections, resulting in a high mortality rate of 40-60%. Here we report a 26-day-old neonate with fever, cough, fast breathing, poor feeding, subcostal retraction, bilateral crackles, hepatomegaly, and signs of shock. Clinically, septic shock and pneumonia were suspected. Respiratory distress worsened despite broad-spectrum antibiotics, and kept getting worse up to the point that intubation and assisted mechanical ventilation were needed. The examination of acid-fast bacteria (AFB) stain on the endotracheal aspirate showed a positive result. The patient was diagnosed as having perinatal TB, then treated with anti-TB regimens (isoniazid, rifampicin, pyrazinamide, ethambutol), as well as prednisone and other supportive medical care. Recurrent septic shock, laryngeal edema, and a possibility of ventilator-associated pneumonia (VAP) worsened the patient’s condition. The patient eventually passed away on the 71^st^ day of care. In conclusion, perinatal TB should be suspected in any neonates with fever, respiratory distress, and hepatosplenomegaly, particularly in those from endemic areas, which fail to respond to broad-spectrum antibiotics. Early diagnosis and treatment are crucial to improve the prognosis of perinatal TB.

## Introduction

Perinatal tuberculosis (TB) is a rare infectious disease caused by *Mycobacterium tuberculosis* [1]. Perinatal TB may be congenital or acquired after birth. Congenital TB is due to transplacental hematogenous spread or ingestion/inhalation of contaminated amniotic fluid or vaginal secretion in the uterus or during childbirth. Neonatal TB may be acquired after birth as a result of ingestion/inhalation of infected droplets or the contamination of skin or mucosal lesions by infected material [1-3]. The diagnosis of perinatal TB is challenging due to its nonspecific clinical manifestations make it difficult to differentiate from other infections, resulting in a high mortality rate of 40-60% [1,4-6]. Therefore, early diagnosis is an essential step in the treatment of perinatal TB [1]. In this paper, we report a case of perinatal TB in a 26-day-old neonate in a remote area of Asmat Regency of South Papua, Indonesia.

## Patient and observation

**Patient information:** a 26-day-old neonate weighing 2,500 grams was referred by the public health center to the emergency room (ER) of Regional Public Hospital of Agats with complaints of fever, cough, tachypnea, and poor feeding since the age of 21 days old. The history of pregnancy and childbirth from the patient´s mother were unclear because she had never done antenatal care during her pregnancy and the delivery was aided by no medical staff.

**Clinical findings:** the patient was lethargic with a temperature of 37.8°C, a heart rate of 180 beats/minute, a respiratory rate of 64 breaths/minute, and an oxygen saturation of 85% in the room air. Physical examination revealed subcostal retraction, bilateral crackles, hepatomegaly, and signs of shock, such as cold extremities, prolonged capillary refill time, and weak pulse.

**Diagnostic assessment:** laboratory results indicated a hemoglobin of 13.4 g/dL, leukocytes of 21,260/mm^3^, platelets of 280,000/mm^3^, blood sugar of 111 mg/dL, sodium of 136 mmol/L, potassium of 2.5 mmol/L, normal urinalysis, and a negative severe acute respiratory syndrome coronavirus 2 (SARS-CoV-2) antigen swab. The patient was hospitalized with diagnosis of septic shock and neonatal pneumonia. The patient was given broad-spectrum intravenous antibiotics, as well as other supportive medical care. A chest X-ray could not be performed due to a technical problem; therefore, a chest ultrasound was performed as an alternative radiological examination. A chest ultrasound discovered a B-line image. The patient experienced a temporary improvement. Unfortunately, clinical deteriorations happened on the 16^th^ day of care. Due to the respiratory distress continued to worsen, intubation was performed and mechanical ventilator assistance was provided. An acid-fast bacteria (AFB) stain and a rapid molecular test were performed on the endotracheal aspirate. A positive result of AFB stain, which was sensitive to rifampicin, was discovered. Based on these findings, the patient was diagnosed as having perinatal TB. Tuberculosis screening was conducted on the patient´s mother with a positive result. Investigation towards the possibility of the human immunodeficiency virus (HIV) showed a negative result on the patient´s mother.

**Therapeutic interventions:** the patient was treated with anti-TB regimens (isoniazid, rifampicin, pyrazinamide, ethambutol), prednisone, along with other supportive medical care.

**Follow-up and outcome of interventions:** recurrent septic shock, laryngeal edema, and the possibility of ventilator-associated pneumonia (VAP) worsened the patient´s condition. The patient eventually passed away on the 71^st^ day of care.

**Informed consent:** written informed consent was obtained from the patient´s mother.

## Discussion

Perinatal TB is a rare disease with only 358 cases reported in the literature up to 1995, 18 cases between 2001 and 2005, and another 21 cases between 2011 and 2017 [7,8]. Although it is considered a rare disease, perinatal TB has a very high mortality rate of up to 40-60%, due to its nonspecific clinical manifestations and frequently mimics other infections, resulting in a high possibility of misdiagnosis [1-3]. The study conducted by Li *et al*. showed that misdiagnosis occurred in almost 60% of cases of perinatal TB. It is most frequently misdiagnosed as pneumonia or sepsis [3]. Fever, hepatosplenomegaly, and respiratory distress are the most common manifestations of perinatal TB, which occur frequently in the second to fourth week of life. Septic shock may develop in severe cases [2-4,6]. As discovered in this case, clinical manifestations appeared in the fourth week of life.

Perinatal TB should be suspected and investigated in the following conditions: (i) neonates who failed to respond to conventional management of pneumonia, particularly in those from endemic areas, (ii) if the mother or family member is diagnosed as having TB, (iii) in the presence of unexplained fever and hepatomegaly [2,9]. In this case, the patient experienced symptoms similar to sepsis and neonatal pneumonia. However, after being treated with conventional management of pneumonia, the patient did not show a good response. In addition, the patient was in Papua, an area with the highest prevalence of TB in Indonesia, up to 0.77%, according to the Indonesian National Basic Health Research Report/*Riset Kesehatan Dasar* (RISKESDAS) 2018. The diagnosis of perinatal TB must be proven with several supporting examinations, such as chest X-ray or other radiological examinations, AFB stain, and TB culture. Suitable specimens for AFB stain and TB culture are gastric lavage, endotracheal aspirate, ascitic, pleural, cerebrospinal fluid, and tissue biopsy [2,4,7]. In contrast to TB in older children, the tuberculin test is not very helpful in neonates because it frequently yields negative results due to an immature immune system [3]. In this case, the diagnosis of perinatal TB was confirmed after a positive result of AFB stain on the endotracheal aspirate.

The investigation on the patient´s mother was performed after the patient was diagnosed as having perinatal TB. The patient´s mother had a mild cough since the second trimester of pregnancy, but was not diagnosed as having TB because she had never had antenatal care. A positive result of AFB stain on the sputum of the patient’s mother was discovered.

Perinatal TB may be congenital or acquired postnatally. Congenital TB can be distinguished from neonatal TB based on the Beitzke criteria in 1935, later revised by Cantwell in 1994. Congenital TB is referred to when a neonate with documented tuberculous lesions satisfies at least one of the following criteria: primary hepatic complex or caseating hepatic granulomas; lesions in the first week of life; tuberculous infection of the maternal genital tract or placenta; and exclusion of postnatal transmission through a careful investigation of contacts [4]. Meanwhile, distinguishing congenital TB from neonatal TB is not crucial, since the treatment and prognosis are similar [6].

In this case, although a chest X-ray could not be performed, we still considered the possibility of miliary TB in the patient by considering the severity of the patient´s disease. Based on this consideration, we gave anti-TB regimens, including isoniazid, rifampicin, pyrazinamide, and ethambutol for two months, which were planned to be followed by isoniazid and rifampicin for seven to ten months. We also administered prednisone according to the recommendation of the Indonesian National Guidelines for TB 2019. Perinatal TB may result in a fatal outcome if left untreated or if the treatment gets delayed [6]. In this case, the patient eventually passed away due to delayed anti-TB regimens administration. Recurrent septic shock, laryngeal edema, and VAP also worsened the patient´s condition.

The limitations, in this case, are due to limited resources. A series of chest X-rays, cultures, and other laboratory tests to exclude the differential diagnoses could not be done.

## Conclusion

Perinatal TB is a rare disease and difficult to differentiate from other infections due to its nonspecific clinical manifestations. Perinatal TB should be suspected in any neonates with fever, respiratory distress, and hepatosplenomegaly, particularly in those from endemic areas, which fail to respond to broad-spectrum antibiotics. Early diagnosis and treatment are crucial to improve the prognosis of perinatal TB.
